# Influence of Coalescence on the Anisotropic Mechanical and Electrical Properties of Nickel Powder/Polydimethylsiloxane Composites

**DOI:** 10.3390/ma9040239

**Published:** 2016-03-29

**Authors:** Sung-Hwan Jang, Yong-Lae Park, Huiming Yin

**Affiliations:** 1 Robotics Institute, School of Computer Science, Carnegie Mellon University, Pittsburgh, PA 15213, USA; ylpark@cs.cmu.edu; 2 Department of Civil Engineering and Engineering Mechanics, Columbia University, New York, NY 10027, USA; yin@civil.columbia.edu; 3 Department of Mechanical and Aerospace Engineering, Seoul National University, Seoul 08826, Korea

**Keywords:** metal-polymer composite, magnetic field, alignment, coalescence, electrical conductivity, mechanical property

## Abstract

Multifunctional polymer-based composites have been widely used in various research and industrial applications, such as flexible and stretchable electronics and sensors and sensor-integrated smart structures. This study investigates the influence of particle coalescence on the mechanical and electrical properties of spherical nickel powder (SNP)/polydimethylsiloxane (PDMS) composites in which SNP was aligned using an external magnetic field. With the increase of the volume fraction of the SNP, the aligned SNP/PDMS composites exhibited a higher tensile strength and a lower ultimate strain. In addition, the composites with aligned SNP showed a lower percolation threshold and a higher electrical conductivity compared with those with randomly dispersed SNP. However, when the concentration of the SNP reached a certain level (40 vol. %), the anisotropy of the effective material property became less noticeable than that of the lower concentration (20 vol. %) composites due to the change of the microstructure of the particles caused by the coalescence of the particles at a high concentration. This work may provide rational methods for the fabrication of aligned composites.

## 1. Introduction

Conductive polymer composites [1,2,3,4,5] have been recently explored and implemented in applications of flexible sensors and sensor-integrated smart structures for structural health monitoring in next generation infrastructures [6,7,8]. Although conventional strain sensors, such as metal-foil or silicon strain gauges, have been widely used for structural health monitoring [9,10], their inherent limitations, such as the requirement of rigid substrates, narrow dynamic ranges, and mechanical fragility, necessitated the development of new types of sensing materials. To overcome these challenges, various types of polymer composites have been proposed using advanced materials, such as ceramics [11,12], ferromagnetic particles [8,13], carbon nanotubes [14,15,16], and embedded liquid conductors [17,18,19]. Chun *et al.* have developed stretchable and flexible conductive films composed of carbon nanotubes and silver flakes for achieving a high conductivity at a high tensile strain (20 S/cm at 140% strain) [20]. Yamada *et al.* have proposed a carbon nanotube film strain sensor that can be incorporated into the human body [21]. They were able to achieve high strains up to 280% with high durability and a fast response. 

In addition to the type of embedded conductive filler particles, the alignment of particles using mechanical, electrical, or magnetic forces is also a useful technique to enhance the overall material properties of a composite [22,23,24,25,26,27]. Since the alignment methods can make the conductive fillers better networked and aligned in a desired direction in the matrix, we can obtain improved performances of the composite in terms of mechanical and electrical properties compared with composites with randomly dispersed fillers. For instance, Song *et al.* have aligned a small amount of ferromagnetic particles in epoxy using a magnetic field for improved mechanical properties of the composite, such as Young’s modulus, tensile, and tear strength [28]. Sun *et al.* have effectively controlled the alignment and the orientation of nickel nanowires in polydimethylsiloxane (PDMS) for anisotropic material behaviors of the composite in both mechanical and magnetic properties [29]. In particular, magnetic alignment has been widely used in polymer-based composites with filler particles [30,31,32]. The chain-structured particle alignments are achieved under an external magnetic field due to mutual interactions between particles that are close to each other. Also, the chain-structured particles may coalesce into columnar structures depending on several factors, such as strength and direction of the external magnetic field, magnetization time, and particle concentrations. The coalescence naturally occurs even in small volume fractions of particles because of particle-chain interactions [33]. However, most of the previous reports limited the concentration of filler particles to relatively small (less than 30 vol. %) [8,34] in the composite, describing only the effect of the particle alignment on the material’s properties, but not taking the effect of coalescence into account. Therefore, to fully characterize the material properties of this type of composite, a further investigation is needed in a wider range of particle concentrations, including high volume fractions over 30 vol. %, considering the relationship between coalescence and volume fraction of filler particles.

In this study, we fabricated conductive polymer composites composed of spherical nickel powder (SNP) and polydimethylsiloxane (PDMS) with different particle concentrations. Different concentrations of SNP were aligned in pre-polymer using an external magnetic field during the curing process. An optical microscope was used to characterize the alignment and the coalescence of the SNP particles. The composites were experimentally characterized to evaluate the effect of the coalescence on the anisotropic material properties with different particle concentrations.

## 2. Experimental

### 2.1. Materials and Sample Preparation

PDMS (Sylgard 184) was obtained from Dow Corning (Midland, MI, USA). PDMS is a non-conductive material with a conductivity of approximately 2.0 × 10^−14^ S/m. Spherical nickel powder (SNP, SNP-400) was obtained from NOVAMET (Lebanon, TN, USA). The average diameter of the SNP is 11.4 μm, and the electrical conductivity is about 1 × 10^6^ S/m. Densities of the PDMS and the SNP are 0.96 g/cm^3^ and 8.65 g/cm^3^, respectively. 

**Figure 1:**
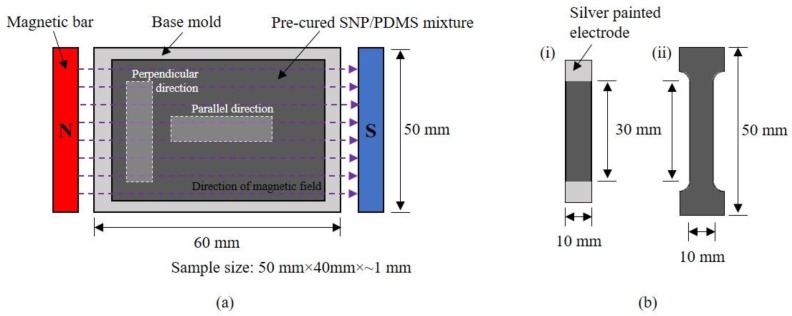
Experimental setup and sample preparation for electrical conductivity and tensile test: (**a**) Experimental set-up; and (**b**) Sample preparation for: (**i**) electrical conductivity and (**ii**) tensile test.

After PDMS and as-received SNP were weighed for the target weight fractions (0, 10, 20, 30, and 40 vol. %), they were mixed using a polymer mixer (ARE 301, Thinky Corp., Tokyo, Japan) at 2000 rpm for three minutes at room temperature in order to obtain a uniform mixture. After dispersion, a curing agent was added to the mixture at a weight ratio of 10:1 for the elastomer and the curing agent, respectively, and mixed again for another three minutes. The mixture was then degassed in vacuum for 10 min to eliminate air voids in the matrix and poured into a mold. An external magnetic field (approximately 1.0 Tesla) was applied to the sample for the alignment of SNP at room temperature, as shown in Figure 1a. Finally, the sample was cured in an oven at 120 °C for one hour. The samples were kept under the magnetic field for continuous alignment during the entire curing process. After fully curing, the samples were lifted off from the mold and post-cured at 60 °C for 1 h.

### 2.2. Characterization

The alignment of SNP in the pre-polymer was characterized using an optical microscope. The direct current (DC) conductivity was measured by a standard two-probe method using two multimeters. For the electrical conductivity, the samples were cut into 10 mm × 30 mm. The electrodes were coated with high-purity silver on both sides of the sample to minimize contact resistances (Figure 1b). Some samples with higher resistances (above 1 GΩ) were measured by a precision multimeter (Keitheley 6517B, Beaverton, OR, USA) and the others with lower resistance (below 1 GΩ) was measured using a regular multimeter (Fluke 8845A, Everett, WA, USA). Then, the electrical conductivity (*σ*) was calculated as following:

(1) $$\sigma =\frac{L}{RA}$$

where *R* is the measured resistance, *A* is the cross-sectional area of the composite; and *L* is the length between the electrodes. Tensile test of the samples was conducted using a motorized materials test stand (ESM301L, Mark-10) at room temperature according to ASTM D412 [35]. The samples were cut in a dog-bone shape in two directions, parallel and perpendicular to the applied magnetic field (Figure 1b). The displacement control was performed and the crosshead velocity was 30.0 mm/min. Five samples of each material were tested for different sample directions (perpendicular and parallel) at room temperature.

## 3. Results and Discussion

**Figure 2:**
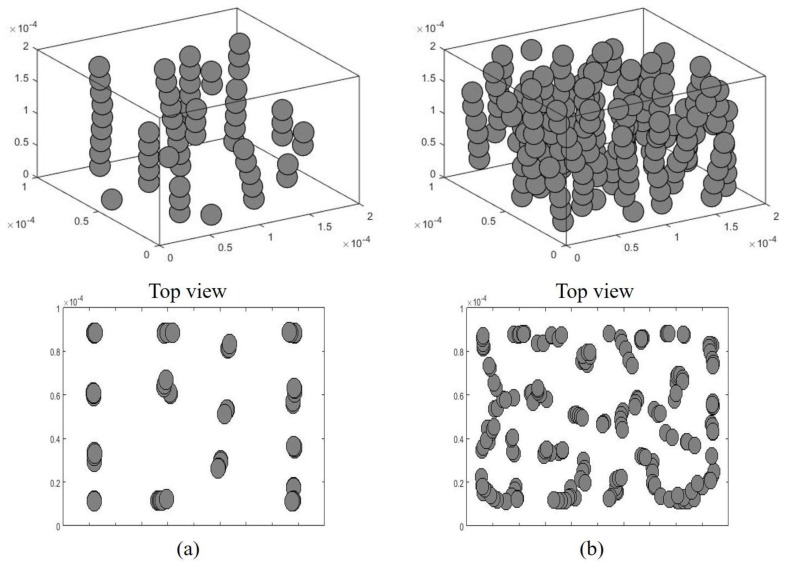
3D simulation results of particle alignments in composite structures; (**a**) 5 vol. % nickel particles; and (**b**) 30 vol. % nickel particles.

Since optical investigation did not make it easy to understand the microstructural change in particles in the pre-polymer in a three-dimensional (3D) space, we used a 3D dynamic simulation for the particle alignment in the pre-polymer state. Figure 2 shows the simulation results of SNP alignment in a 3D space with two different particle concentrations. Although the movement of magnetic particles is affected by different factors, such as the magnetic force, van der Waals force, and gravitational and buoyancy forces, the simulation for the particle alignment was established using Newton’s second law of motion. Total forces include the Stokes’ drag force, dipole-dipole interaction force, and repulsive force due to contact with other particles and with walls. The simulation was conducted using the Runge-Kutta algorithm. More details in the simulation can be found in [36]. In this study, we simulated 30 vol. % of particles for a high volume fraction due to the computation time.

**Figure 3:**
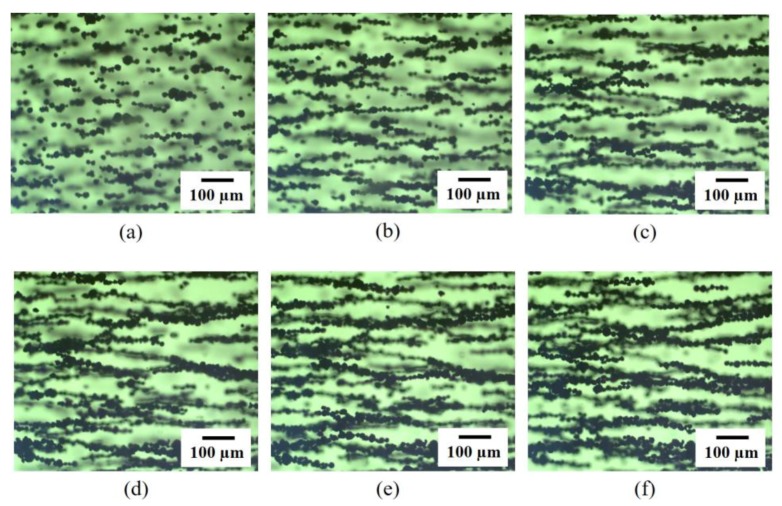
Alignment process of SNP in the matrix (10 vol. %) with magnetization times: (**a**) 1 second; (**b**) 5 seconds; (**c**) 10 seconds; (**d**) 15 seconds; (**e**) 20 seconds; and (**f**) 25 seconds.

**Figure 4:**
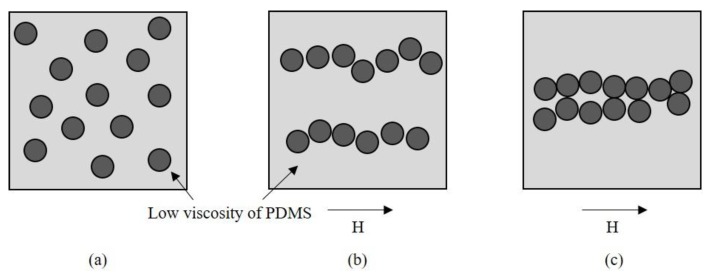
Illustration of particle alignment process in the pre-polymer state: (**a**) Without magnetic field; (**b**) Magnetic field with short magnetization time; and (**c**) Magnetic field with long magnetization time.

For the low volume fraction of particles, as shown in Figure 2a, relatively clear chain-like particle structures were observed without any interaction between other chains. Figure 3 shows experimental results for the alignment process of the SNP in the matrix according to the magnetization time. When the external magnetic field was applied, the SNP was quickly aligned in parallel with the direction of the magnetic field, forming a short-chain structure, and finally making large aggregates with the magnetization time. This occurred due to the magnetization of the particles, forming a chain structure resulting in thick aggregates [37,38]. Figure 4 shows an illustration of magnetically induced phase transition with magnetization time. Randomly dispersed particles exist in the pre-polymer state before applying the magnetic field, as shown in Figure 4a. When the magnetic field is strong enough, the magnetic moment of the particle reaches a saturated value, and the dipole-dipole interaction force drives particles into chain-like structures along the dipole moment, as shown in Figure 4b. Then, when inter-chain repulsive forces are strong enough, the system becomes thermodynamically unstable and the chains start to aggregate into different shapes, such as zigzag multi-chains, as shown in Figure 4c [39]. 

**Figure 5:**
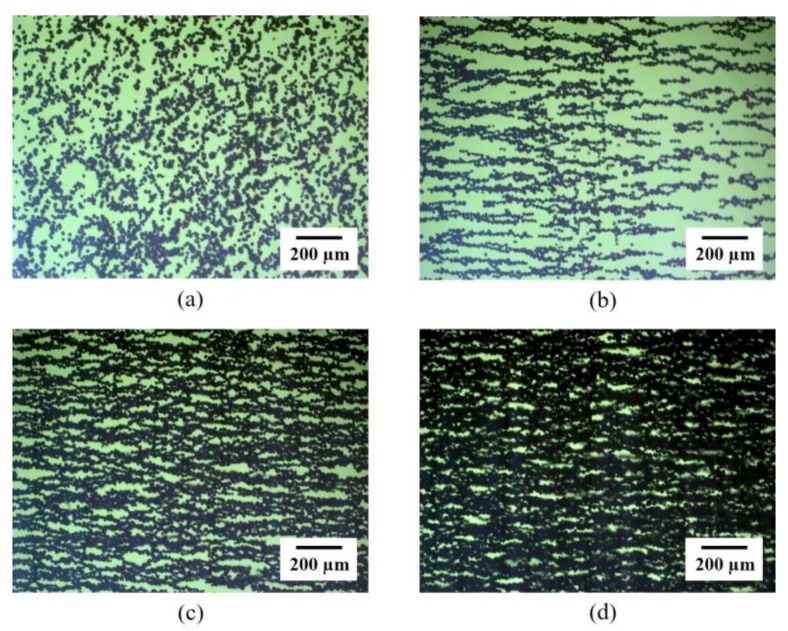
OM images of SNP in the matrix at different concentration; (**a**) 10 vol. % SNP without magnetic field; (**b**) 10 vol. % SNP under magnetic field; (**c**) 20 vol. % SNP under magnetic field; and (**d**) 30 vol. % SNP under magnetic field.

On the other hand, the nickel particles formed lateral coalescence in some places with a higher concentration, as shown in Figure 2b. Figure 5 presents microscopic images of the SNP in the matrix with different volume fractions with and without the magnetic field. Figure 5a shows randomly dispersed SNPs in the matrix without applying the magnetic field. With the magnetic field, nickel particles were quickly aligned in parallel (Figure 5b). The alignment was clearly observed until the volume fraction reached 20 vol. % (Figure 5c). However, it was difficult to identify the alignment pattern with a high volume fraction (Figure 5d) since the average inter-particle distance became smaller as the particle concentration increased. Moreover, the magnetization of the SNP may also have attracted the particles to each other, resulting in the rearrangement of the microstructure of the aligned particles to aggregation in the matrix during the curing process. Therefore, at a high volume fraction, the particle rearrangement into complex structures was observed under the same external magnetic field. This change in microstructure may have affected overall material properties such as electrical and mechanical properties of the aligned SNP/PDMS composite. 

**Figure 6:**
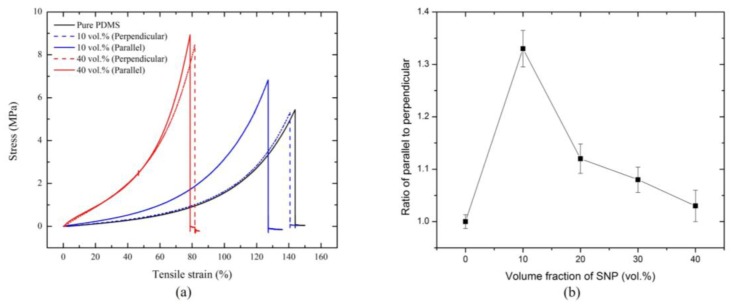
(**a**) Stress-strain curve of tensile strength testing for pure PDMS and SNP/PDMS composites; and (**b**) tensile stress ratio of parallel to perpendicular SNP configurations.

Now, we investigated the influence of SNP coalescence on the mechanical properties of the SNP/PDMS composites. Figure 6a shows three representative stress-strain curves of the SNP/PDMS composite samples with different SNP concentrations from experimental measurements. In general, pure PDMS cured at a high temperature ruptures at a lower tensile strain than that cured at room temperature [40]. When the SNP concentration was low (10 vol. % of SNP), the SNP/PDMS composite clearly showed anisotropy in the stress-strain curve. In our results, while the composite perpendicular to the magnetic field showed a similar stress-strain characteristic to that of pure PDMS, the composite parallel to the magnetic field showed a higher tensile stress at a less tensile strain. However, when the SNP concentration was significantly increased (40 vol. % of SNP), the composite showed an almost isotropic behavior regardless of the direction of the applied magnetic field, as shown in Figure 6b. Table 1 summarizes the averaged maximum tensile strengths and strains of five SNP/PDMS composite samples with varied SNP volume fractions. It was observed that the maximum tensile strain of SNP/PDMS composites dramatically decreased with increased SNP volume fractions. Although the SNP/PDMS composite samples exhibited lower stretchability than pure PDMS, all four samples were able to stretch more than 10% of their original lengths, which confirmed that SNP/PDMS composites are suitable for detecting strain changes of infrastructures for structural health monitoring.

**Figure 7:**
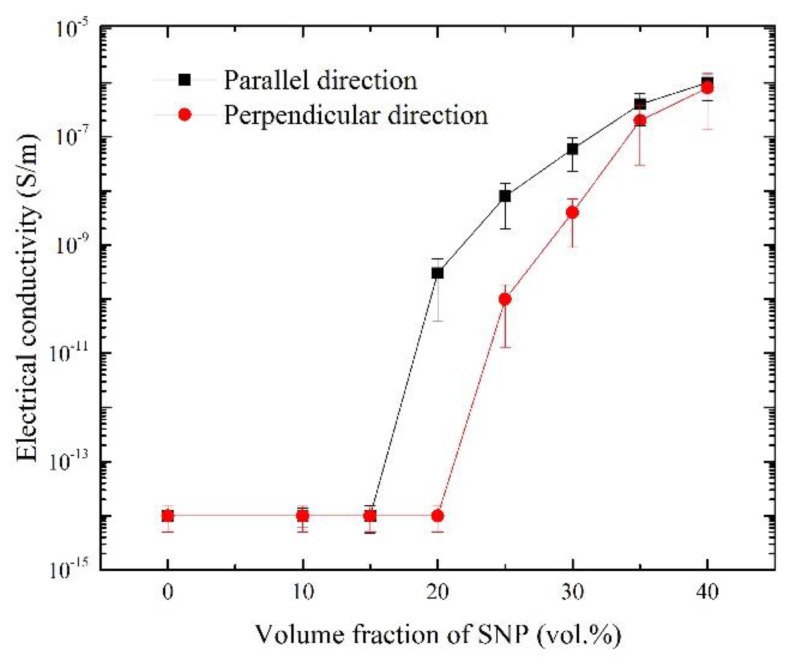
Electrical conductivity of SNP/PDMS composite.

In addition to the mechanical property, the electrical property of the SNP/PDMS composites was also characterized. Figure 7 shows the electrical conductivity of pure PDMS and SNP/PDMS composites as a function of SNP concentrations. Table 2 summarizes the electrical conductivity of the SNP/PDMS composites with different SNP concentrations and the directions relative to the SNP chains. The electrical conductivity of pure PDMS is approximately 10^−14^ S/m, showing no change in electrical conductivity up to 20 vol. % of SNPs regardless of the direction. This means that the SNPs do not make a conductive network in the matrix. Based on the percolation theory, the electrical conductivity of composites is mainly dependent on the volume fraction, the size, the shape of the fillers, and the impurity [41,42,43]. Note that the fillers in our samples were micron-size particles with spherical shapes that affected the lower electrical conductivity. However, in this study, the SNP was used to understand the influence of its coalescence and alignment on the anisotropy of a composite material and its electrical conductivity.

Rapid increases in electrical conductivity were observed with SNP/PDMS composites between 15 vol. % and 20 vol. % and between 20 vol. % and 25 vol. %, in parallel with and perpendicular to the magnetic field, respectively. This phenomenon can be explained because the aligned SNP in the matrix enhanced the conductivity of the SNP network due to the alignment process under the magnetic field. However, the SNP/PDMS composites at above 35 vol. % showed similar electrical conductivities in both directions (*i.e.*, parallel and perpendicular) regardless of the magnetic field. This effect may be attributed to the morphology change in SNP in the matrix as observed in the experiment. The loss of anisotropy in electrical conductivity can be explained by the loss of anisotropy in the distribution of the SNP in the matrix due to high particle concentration.

## 4. Conclusions

The main contribution of this work is to examine the influence of coalescence of nickel particles on the mechanical and electrical properties of SNP/PDMS composites. Although conductive polymer composites have been proposed and tested for various smart composite structures, relatively small concentrations of filler particles have been taken into account in most cases, which explains only the effect of the alignment of the filler particles. In our study, the alignment of SNPs in the matrix was achieved by an external magnetic field as a function of particle concentrations up to 40 vol. %. Visual inspections and dynamic simulation confirmed the alignment of SNP and the coalescence process with time as well as volume fractions of SNP. SNP-aligned composites showed significant anisotropy in both mechanical and electrical properties depending on the volume fractions. While the anisotropy material properties were clearly observed at low SNP concentrations in terms of the mechanical and electrical properties, the material properties were changed from anisotropic to isotropic with an increase of the SNP concentration, which is due to the magnetization of the filler particles, forming the coalescence of nickel particles and making structural rearrangements. In addition, the effects of the alignment of SNP were maximized at 10 vol. % and 20 vol. % SNP/PDMS composites for mechanical and for electrical anisotropies, respectively. This work provides a practical understanding of mechanical and electrical properties of conductive polymer composites with magnetically aligned filler particles influenced by the coalescence of particles. The SNP/PDMS composite can be used as smart composites to monitor electrical and/or thermal properties, and thus it is important to show a stable signal response under external loading [44,45,46,47]. The future research will include the long-term stability of the SNP/PDMS composite under cyclic loadings for practical applications.

## Figures and Tables

**Table 1:** Maximum tensile strength and strain of PDMS and SNP/PDMS composites.

Sample	Ultimate Tensile Strength (MPa)	Maximum Tensile Strain (%)
Pure PDMS	5.39 ± 1.23	144 ± 9.3
10 vol. % (Perpendicular)	5.11 ± 1.53	141 ± 10.2
10 vol. % (Parallel)	6.78 ± 1.52	127 ± 9.1
20 vol. % (Perpendicular)	6.90 ± 1.42	137 ± 11.5
20 vol. % (Parallel)	7.48 ± 1.48	120 ± 10.2
30 vol. % (Perpendicular)	6.98 ± 1.30	97 ± 9.1
30 vol. % (Parallel)	7.80 ± 1.02	89 ± 9.4
40 vol. % (Perpendicular)	8.23 ± 1.21	82 ± 8.8
40 vol. % (Parallel)	8.43 ± 1.43	79 ± 10.3

**Table 2:** Summary of electrical conductivity of SNP/PDMS composite.

Volume Fraction of SNP (vol. %)	Electrical Conductivity (S/m)		Ratio of Parallel to Perpendicular
	Perpendicular	Parallel	
10	1.0 ± 0.04 × 10^−14^	1.0 ± 0.05 × 10^−14^	1.0
20	1.0 ± 0.05 × 10^−14^	3.2 ± 0.03 × 10^−10^	32,000.0
30	4.4 ± 0.03 × 10^−9^	6.1 ± 0.04 × 10^−8^	13.9
40	8.2 ± 0.07 × 10^−7^	1.3 ± 0.05 × 10^−6^	1.6
